# Supplementary material for: Spatiotemporal gait characteristics post-total hip arthroplasty and its impact on locomotive syndrome: a before-after comparative study in hip osteoarthritis patients
Source: PeerJ. 2024 Oct 22;12:e18351. doi: 10.7717/peerj.18351 (PMC11505882; doi:10.7717/peerj.18351)
Supplement: File S2 [file peerj-12-18351-s002.docx]

Codebook

- Sex

1: male
2: female

- Standuplocomo

CDL stages of the stand-up test before THA

0: No locomotive syndrome
1: Stage 1
2: Stage 2
3: Stage 3

- Standupscore

Performance scores of the stand-up test before THA

0: Score 0

1: Score 1
2: Score 2
3: Score 3
4: Score 4

5: Score 5

6: Score 6

7: Score 7

8: Score 8

- TwostepScore

2-step values before THA

- Twosteplocomo

CDL stages of the two-step test before THA

0: No locomotive syndrome
1: Stage 1
2: Stage 2
3: Stage 3

- GLFS_25

GLFS-25 scores before THA

- GLFS_25locomo

CDL stages of the GLFS-25 before THA

0: No locomotive syndrome
1: Stage 1
2: Stage 2
3: Stage 3

- TotalLocomo

Total CDL stages before THA

0: No locomotive syndrome
1: Stage 1
2: Stage 2
3: Stage 3

- OPSCadence

THA side cadence before THA

- NOPSCadence

Non THA side cadence before THA

- OPS_StrideTime

THA side stride time before THA

- NOPS_StrideTime

Non THA side stride time before THA

- OPtoNOPStepTime

THA side to Non THA side step time before THA

- NOPtoOPStepTime

Non THA side to THA side step time before THA

- OPSSingleSupport

THA side single support time before THA

- NOPSSingleSupport

Non THA side single support time before THA

- OPStoNOPSDoubleSupport

THA side to Non THA side double support time before THA

- NOPStoOPSDoubleSupport

Non THA side to THA side double support time before THA

- OPSStrideLength

THA side stride length before THA

- NOPSStrideLength

Non THA side stride length before THA

- OPtoNOPStepLength

THA side to Non THA side step length before THA

- NOPtoOPStepLength

Non THA side to THA side step length before THA

- OPSWalkingSpeed

THA side walking speed before THA

- NOPSWalkingSpeed

Non THA side walking speed before THA

- StanduplocomoA

CDL stages of the stand-up test three months after THA

0: No locomotive syndrome
1: Stage 1
2: Stage 2
3: Stage 3

- StandupScoreA

Performance scores of the stand-up test three months after THA

0: Score 0

1: Score 1
2: Score 2
3: Score 3
4: Score 4

5: Score 5

6: Score 6

7: Score 7

8: Score 8

- TwostepScoreA

2-step values three months after THA

- TwoSteplocomoA

CDL stages of the two-step test three months after THA

0: No locomotive syndrome
1: Stage 1
2: Stage 2
3: Stage 3

- GLFS25A

GLFS-25 scores three months after THA

- GLFS_25locomoA

CDL stages of the GLFS-25 three months after THA

0: No locomotive syndrome
1: Stage 1
2: Stage 2
3: Stage 3

- TotalLocomoA

Total CDL stages three months after THA

0: No locomotive syndrome
1: Stage 1
2: Stage 2
3: Stage 3

- OPSCadenceA

THA side cadence three months after THA

- NOPSCadenceA

Non THA side cadence three months after THA

- OPS_StrideTimeA

THA side stride time three months after THA

- NOPS_StrideTimeA

Non THA side stride time three months after THA

- OPtoNOPStepTimeA

THA side to Non THA side step time three months after THA

- NOPtoOPStepTimeA

Non THA side to THA side step time three months after THA

- OPSSingleSupportA

THA side single support time three months after THA

- NOPSSingleSupportA

Non THA side single support time three months after THA

- OPStoNOPSDoubleSupportA

THA side to Non THA side double support time three months after THA

- NOPStoOPSDoubleSupportA

Non THA side to THA side double support time three months after THA

- OPSStrideLengthA

THA side stride length three months after THA

- NOPSStrideLengthA

Non THA side stride length three months after THA

- OPtoNOPStepLengthA

THA side to Non THA side step length three months after THA

- NOPtoOPStepLengthA

Non THA side to THA side step length three months after THA

- OPSWalkingSpeedA

THA side walking speed three months after THA

- NOPSWalkingSpeedA

Non THA side walking speed three months after THA
